# Supplementary material for: What sustain Chinese adult second language (L2) learners’ engagement in online classes? A sequential mix-methods study on the roles of L2 motivation and enjoyment
Source: PLoS One. 2025 Jan 24;20(1):e0317761. doi: 10.1371/journal.pone.0317761 (PMC11759370; doi:10.1371/journal.pone.0317761)
Supplement: S1 File — (ZIP) [file pone.0317761.s001.zip › ORIGINAL DATA/INTERVIEW DATA.docx]

**Interviewee 1**

**Interviewer:** What factors motivate you to be engaged in the online second language class? Could you describe them in detail？

**Interviewee 1:**

Well, when it comes to staying focused in online second-language courses, I have to say, it's not something that happens overnight. First of all, I have to admit that for me, English is really more than just a work tool. I used to think that once I was in the system, English didn't seem to have much use, but after a few years of working, I gradually realized that learning English well can actually bring a lot of unexpected benefits.

I studied English with the goal of future educational advancement. Initially, I, along with others in the government sector, doubted the practicality of learning English due to its limited use. However, my seven years of experience there revealed the unexpected advantages of English proficiency. For anyone eyeing an MPA or part-time graduate studies, or a full-time academic return, English is an essential exam component. I've noticed many colleagues struggle with English exams due to post-work language disuse, which hampers their preparation. Post-admission, the stringent demands of academic and master's English courses add to the challenge.

Additionally, I've heard that there are quite a few opportunities for overseas training or exchanges within the system, and these opportunities sound very appealing. A friend of mine who works in a central government agency told me that their organization has fully-funded slots for studying abroad at world-renowned universities, with your salary continuing, position retained, and tuition waived. But the key is, you need to have IELTS or TOEFL scores. So, when I study, I think about these opportunities, feeling that I need to try harder.

Moreover, I feel that learning English helps me maintain an open mindset. I really dislike rigid and inflexible thinking, so while adapting to the system, I also hope to broaden my horizons through learning English. For me, English is like a key that allows me to access higher quality information sources and see more of the world.

Through online courses, I've met many interesting people. Some work at international schools, others study abroad, and their lives and ideas are all very fresh to me. I've seen the changes in the world through them and learned about many trendy things. Also, I've encountered some older students who need English to boost their careers or lives, which makes me feel that our connection with the world is becoming increasingly tight.

Interacting with them, I've not only learned English but also gained new perspectives and inspirations for life and work. For example, designers from major internet companies, how their design philosophies are similar to my teaching philosophies, and what insights can be borrowed. There are also business students who can share core methods of interpersonal communication to improve communication efficiency. With students in technology, I can exchange insights on skill learning.

It can also be about life experiences, such as the difficulties an older student might face when starting to learn a new language, the emotional journey of going to a new country, and the challenges of balancing all aspects of life. Although I haven't experienced these directly, I've been able to glimpse them through them. These rare experiences are brought to me by this profession and are also opportunities given to me by persisting in learning English.

Learning English has brought me more than just improved language skills; it's a new way of understanding the world. It gives me the chance to meet people from different countries and fields, bringing me many new perspectives and inspirations. These are the reasons why I stay focused in online second-language courses. English is like a special door that allows me to open up the world from a new angle, bringing me a lot of growth and joy.

**Interviewer:** Thank you for sharing with us. I truly appreciate your generous cooperation.

**Interviewee 2**

**Interviewer:** What factors motivate you to be engaged in the online second language class? Could you describe them in detail？

**Interviewee 2:**

Why am I so focused in class? Because I really enjoy learning English. Actually, my motivation for learning English is not driven by utilitarianism, such as finding a good job or obtaining a higher degree. During my school years, I pondered why ancient China, once so powerful, fell behind Western countries in modern times. I didn't want to find the answer in textbooks. I wanted to visit those countries to understand the cultures of the Western powers. With English as the lingua franca, mastering it is incredibly important. That's why I excelled in English during my school years.

Now that I'm working, I still learn English, but I no longer think about political issues. I simply love the feeling of learning English and the Western culture behind it. Every time I study English, I genuinely feel happy, as if I'm going to a party and my mood is fantastic. When I practice speaking, I get so excited I feel like I'm flying. Memorizing vocabulary is also interesting; using apps to pass levels one by one gives me a great sense of achievement, which motivates me to study more. When listening to English songs, I sing along with the chorus, completely immersed, and my happiness soars.

When looking at engineering materials, if others can't understand but I can, I'm particularly delighted, feeling like I'm reading an encrypted file, which is quite mysterious. Watching American or British TV shows, the plots are so captivating that I can't help but keep watching. I believe learning English allows me to see a broader world and the beauty beyond my life, so now I have a seed planted in my heart that wants to study abroad. When chatting with people who understand English, we can use abbreviations, as if we're having an encrypted conversation, which is particularly fun and increases that strange rapport between us.

Moreover, courses on the history of British and American literature are really super fun and interesting. Perhaps influenced by the Western culture represented by English, I'm less anxious about my appearance now, no longer blindly pursuing thinness. I'm a man, I eat when I should eat, and drink when I should drink. My aesthetics, health consciousness, and values have all changed to some extent, and I feel like I'm getting better at taking care of myself, able to accept my imperfections calmly, and becoming more confident. Learning English also helps me keep a clear mind, constantly reminding me that I don't belong to remote places; there's a broader, more colorful world out there waiting for me to explore and adventure. Once I've learned enough, I definitely want to achieve my dreams.

In short, learning English makes me happier, more fulfilled, and broadens my horizons. I've also made many foreign friends and English experts. Lately, I've been reflecting a lot. I always say I want to be myself, but what does it mean to be myself? I've thought it through, and being myself should at least make me happy, right? So, I'll just do what makes me happy; there's no need to hesitate!

There are definitely many more reasons why I like learning English, but I can't think of them all at once, so I'll let time uncover them slowly. I tell myself to pay more attention to myself, to receive information related to me, and most of the outside information is irrelevant to me, so I don't need to care too much. Don't waste time on an ignorant self, don't be too eager for quick success, take one step at a time, work hard towards your goals, do what you should do at what age, love yourself, slow down, and you'll achieve more with less effort.

**Interviewer:** I’m deeply grateful for your willingness to share with us. Your generous collaboration is sincerely appreciated!

**Interviewee 3**

**Interviewer:** What factors motivate you to be engaged in the online second language class? Could you describe them in detail？

**Interviewee 3:**

What keeps me focused? It's English, truly a powerful tool for my work and the key to opening the door to a new world. Being in the foreign trade business has introduced me to many interesting people who have inspired me greatly.

Chatting with these folks, I get to see a variety of lifestyles, such as how international schools conduct classes, what student life is like abroad, what courses and activities they have, and how their educational philosophies differ from ours. All of this has deepened my understanding of the world. Moreover, through them, I can keep up with the latest trends, language habits, and lifestyles from different places, which makes my job particularly fascinating.

The clients I interact with are often around my age or even older, like those who are still working hard in their careers at 50. We all share one thing in common: we rely on English to advance our careers or improve our quality of life. I've come to realize deeply that in this increasingly globalized world, English has become a bridge connecting the world. These adult learners are striving to overcome language barriers to secure more job opportunities and a better life.

My exchanges with them go beyond just learning; they involve intellectual collisions. For instance, I discuss design concepts with designers from major internet companies to see how their ideas align with or differ from my work philosophy and what I can learn from them. Business-oriented clients share their insights on interpersonal communication and effective interaction. I value these experiences greatly.

Sharing about life is equally important. For example, clients might talk about the challenges of learning a new language or their experiences moving to a new country, as well as the various challenges they face in life. Although I haven't experienced these things firsthand, listening to their stories allows me to indirectly experience those moments. These invaluable experiences are brought to me by my job and are opportunities I've gained by persisting in learning English.

Learning English allows me to understand the world from different perspectives and has changed the way I think. More importantly, it has given me the chance to connect with people from different countries and fields, bringing new viewpoints and inspiration to my life and work. Continuing to learn English has led me to a career that I love and can develop sustainably.

Moreover, learning English has tremendous benefits for my children's education. Nowadays, kids need to broaden their horizons, and English is a must-have skill. Through my own learning experiences, I can better guide them, giving them a good start in language from a young age. This way, they will be more adept in both learning and working in the future.

I can also share with them the joy and importance of learning English, encouraging them to actively explore and enjoy the process rather than just passively learning. This makes their attitude towards learning English more positive, and naturally, the results improve.

More importantly, I can inspire them with my own experiences of learning English, showing them the importance of persistence and effort. I hope they can learn from me that no matter what difficulties they encounter, as long as they keep going, they will see results. This positive attitude is greatly beneficial to their lives.

So, learning English is not only a ladder for my personal growth but also a great help in educating my children. Seeing them make progress bit by bit, the joy in my heart is indescribable. This also strengthens my determination to continue learning English well. For me, English is like a special door that allows me to explore the world from a new angle, bringing me endless growth and joy.

**Interviewer:** Thank you for generously sharing your personal experiences in English learning. It's truly enlightening. I extend my best wishes for your ongoing success.

**Interviewee 4**

**Interviewer:** What factors motivate you to be engaged in the online second language class? Could you describe them in detail？

**Interviewee 4：**

Oh, when it comes to why I'm so dedicated to learning French, it's not something that can be explained in just a few words. It really starts with my own experiences. French means a lot to me, which is why I can focus on learning it.

To be honest, I really hated French when I was a kid, or rather, I had a ton of grievances against it until I went to study in France. When I was little, I was put into a French class where French was the first language. Because of French, I missed out on way more opportunities than I gained. I couldn't participate in English competitions, I couldn't go to Model United Nations, there were no practice questions to brush up on, a lack of listening and reading materials, dictionaries weren't very helpful, and it was hard to find tutoring to improve my score, not to mention original books were impossible to buy. Over a decade ago when I first started learning French, even a decent French assistant was hard to come by. And because of French, no matter how well I scored on my middle school exams, I couldn't go to those top high schools that didn't teach foreign languages, I couldn't get into autonomous recruitment, and during the recommendation exams, good schools either didn't offer French majors or didn't accept students with high-level minority languages, so I could only continue with French. In short, there were just too many regrets.

When I started learning French, I was too young, and it was entirely my parents' decision. Over the years, I've felt countless times how being tied down by French was so beyond my control, so I swore that all the decisions in my life from then on had to be made by me. Choosing subjects, selecting schools, going abroad, every decision after that, I wouldn't leave the choice to anyone else.

I really started to enjoy the benefits of learning French after graduating from high school and dropping out of my undergraduate studies to go abroad. First of all, if it weren't for French, my family would never have been willing to spend millions to send me to mainstream English-speaking countries for both undergraduate and master's degrees. Whether I took the college entrance exam or was recommended, I would have had to finish my undergraduate studies in China before considering studying abroad. Because I knew French, after graduating from high school and passing the DALF C1, I was able to directly apply to my dream school's finance program without taking a foundation course or a language class, saving as much money as possible. Because of French, I was able to go to Paris, the place I've always wanted to go, earlier than planned during my undergraduate studies and live there for a longer time.

At a high-value school with decent rankings, low costs, lots of studying, and a tough life, I earned my French literature diploma and a whole lot of life experiences. Because of French, the summer after my sophomore year, I went to Spain as a French and Chinese tutor, spending a month traveling around Spain. I interned at the Cannes Film Festival, walked the red carpet, and wrote reports. I volunteered on a farm in Marseille, where I got room and board for carrying wood, and I took care of a super kind old lady in Rennes. When I left, she sold me her own Chanel vintage belt for five euros as a souvenir. During my studies abroad, I visited over thirty countries, also because being based in France with a student residence permit allowed me to travel cheaply through the Schengen countries.

After returning to my home country, the job market was very bleak due to the pandemic, and employers started to cut salaries and exploit workers. In a fit of anger, I quit my job and found a relatively easy clerk position with a lower salary but weekends off. I used those weekends to do part-time French tutoring, which made my income quite considerable. It even made more money than when I was working in finance. I have to say, choosing French was the best decision I ever made. It's what backed me up when I couldn't stand the workplace exploitation and decided to quit. It's what gave me the confidence to resign without asking my family for money for nearly half a year and still ensure a five-digit savings account. It's the protective shield when my abilities are limited.

My feelings towards French are complicated, but after all the twists and turns, this language has become my livelihood. French has been infinitely tolerant of me, tolerating my disdain for her, tolerating my attempts to break free from her. So now I have an almost obsessive affection for French. I won't give up this language just as she pulled me up when I was down. When I was struggling the most at work, it was French that helped me find my worth. Because of these letters, I could escape the despair of work.

What makes me focus? Because learning a language is more often not a dead end but a way out. After hitting walls and being beaten down by society, you turn around to find her waiting for you. In her eyes, you always have value, and that's an affirmation worth more than gold.

**Interviewer:** I sincerely appreciate your thorough explanation. I sincerely hope that your life continues to improve and flourish.

**Interviewee 5**

**Interviewer:** What factors motivate you to be engaged in the online second language class? Could you describe them in detail?

**Interviewee 5:**

Haha, I focus because I have to learn it. When it comes to learning English, I used to be completely confused. Many people asked me, "If you're not going abroad, not working for a foreign company, and you'll hardly meet any foreigners in your life, what's the use of learning English?" I used to think the same way, feeling that learning English was just a waste of time.

But later, I gradually understood that there is actually a very practical reason for learning English. That is, I'm focused on accessing information Does that sound a bit unexpected? I have to tell you, I'm not an English major; I'm a computer science major. Back in school, I didn't think English was a big deal. Although I heard people say that English is very important, possibly more useful than many things you learn in college, I just didn't feel it at the time.

I've come to understand the significance of English in my career, which is crucial for my computer science background, as programming languages and tools predominantly originate from Western countries. When facing technical challenges, the most reliable solutions and cutting-edge technologies are often documented in English. Relying on translations can result in inaccuracies and additional costs, whereas English resources are freely available. To bridge the information gap and stay competitive,

It was then that I realized, if I want to elevate my skills to the next level, I can't avoid the hurdle of English. Otherwise, I'd have to wait for others to translate, eating the leftovers of what others have chewed. Those second-hand pieces of information are not only prone to errors, but some even cost money. Yet, the original English sources are all free. This is the information gap. To avoid this gap and obtain first-hand information, one must learn English well.

In fact, it's not just in the computer industry; in most industries, the latest technologies and knowledge are presented in English. After all, the West is still the main source of innovation. Even if many innovations come from non-English speaking countries, they publish their articles in English.

I've digressed a bit. I know many people stop learning and stop pursuing progress once they graduate from college. What do those innovative, cutting-edge things have to do with them? They don't want to improve themselves either.

Indeed, just like the educational philosophy of our country, it merely provides everyone with an equal opportunity. Not everyone who goes to school becomes talented, but as long as someone seizes the opportunity and changes their destiny, that's enough. The same goes for learning English; it may indeed be useless for 90% of people.

This is the main reason I want to learn English: to bridge the information gap and improve myself. I believe it's not just about learning a language; it allows me to access a broader world and gives me the chance to keep up with the times.

**Interviewer:** We are truly grateful for your kindness in offering such an impressive and detailed explanation. It has been immensely helpful for our studies.
