# Supplementary material for: What sustain Chinese adult second language (L2) learners’ engagement in online classes? A sequential mix-methods study on the roles of L2 motivation and enjoyment
Source: PLoS One. 2025 Jan 24;20(1):e0317761. doi: 10.1371/journal.pone.0317761 (PMC11759370; doi:10.1371/journal.pone.0317761)
Supplement: Attachment — Submitted filename: Response to Reviewers for PLOS ONE.docx [file pone.0317761.s002.docx]

**Title: What sustain Chinese adult second language (L2) learners’ engagement in online classes? A sequential mix-methods study on the roles of L2 motivation and enjoyment**

**Responses to Reviewer 1**

**Comments to the Author(s):**

The paper's topic has certain theoretical and practical significance. The overall design is well-executed, aligning with the paradigm and approach of mixed research design. The structure of the paper is complete, and the quantitative and qualitative data effectively address the research questions. However, there are several issues that the authors should consider.

Dear Reviewer 1,

Thank you for your insightful feedback and patience in reviewing my manuscript. We have carefully addressed each of your constructive comments and made the necessary revisions. We hope the updated manuscript meets your approval. We’ve highlighted the revised parts in yellow for your convenience.

Best wishes

**Comment 1:** The grasp of prior literature for this research needs to be strengthened. Please consider the following references

1. Liu, H., Zhong, Y., Chen, H., & Wang, Y. (2023). The mediating roles of resilience and motivation in the relationship between students’ English learning burnout on engagement: A conservation-of-resources perspective. *International Review of Applied Linguistics in Language Teaching*. https://doi.org/10.1515/iral-2023-0089.

2. Wang, X. & Liu, H. (2024). Exploring the moderation roles of emotions, attitudes, environment, and teachers on the impact of motivation on learning behaviors in students’ English learning. *Psychological Reports*, online publication, 1-27. https://doi.org/10.1177/00332941241231714

3. Wang, Y. & Liu. H. (2022). The mediating roles of boredom and buoyancy in the relationship between autonomous motivation and engagement among Chinese senior high school EFL learners. *Frontiers in Psychology*. 13:992279, 1-12. https://doi.org/10.3389/fpsyg.2022.992279

4. Liu, H. & Fan, J. (2024), AI-mediated communication in EFL classrooms: the role of technical and pedagogical stimuli and the mediating effects of AI literacy and enjoyment. *European Journal of Education*. e12813. https://doi.org/10.1111/ejed.12813

5. Liu, H., Zhu, Z., & Chen, B. (2024). Unraveling the mediating role of buoyancy in the relationship between anxiety and EFL students’ learning engagement. *Perceptual and Motor Skills*. https://doi.org/10.1177/00315125241291639

6. Liu, H., Li. J., & Fang. F. (2022). Examining the complexity between boredom and engagement in English Learning: Evidence from Chinese high school students. *Sustainability*, 14, 16920: 1-12. https://doi.org/10.3390/su142416920.

7. Liu, H. & Song, X. (2021). Exploring “Flow” in young Chinese EFL learners’ online English learning activities. *System*, 96, 102425: 1-13. https://doi.org/10.1016/j.system.2020.102425.

Please note you are not obliged to include any of the citations above. Not including the suggested

references have no negative impact on the final editorial decision. You are requested to determine

if the citations add value to the current submission and are free to exclude any/all citations as

appropriate.

**Reply:** Thanks for recommending so many excellent relevant works. I mean why not? These articles are so great and closely related to our study. We’ve included all of these study to our manuscript. Besides, we’ve added more citations to reinforce our literature review:

Liu, H., Elahi Shirvan, M., & Taherian, T. (2024). Revisiting the relationship between global and specific levels of foreign language boredom and language engagement: A moderated mediation model of academic buoyancy and emotional engagement. *Studies in Second Language Learning and Teaching*. <https://doi.org/10.14746/ssllt.40195>

Liu, H. (2024). Demystifying the relationship between parental investment in learners’ English learning and learners’ L2 motivational self system in the Chinese context: A Bourdieusian capital perspective. *International Journal of Educational Development*, *104*, 102973. <https://doi.org/https://doi.org/10.1016/j.ijedudev.2023.102973>

**Comment 2:** Figures should use standardized models instead of non-standardized ones. Additionally, some correlation coefficients are not clear from the figures, such as the relationship between IL2S and several other variables.

**Reply:** Thank you for your comment. In fact, all the figures in our manuscript used standardized models. However, it’s possible that the presentation of our models, especially Figure 2, was unclear, making them appear non-standardized. Additionally, as you mentioned, the correlations between the variables in Figure 2 were not clearly presented. To address this, we have uploaded a clearer version of Figure 2 (The final modified measurement model), as shown below:

**Comment 3:** The authors are requested to check whether the factor loading from engagement to cognition being 1.01 is abnormal, which leads back to the question above about whether non-standardized figures were used.

**Reply:** Thank you for your insightful comment. We also found it quite unusual that the standardized factor loading from engagement to cognition was 1.01. Upon reviewing the data, we hypothesize that multicollinearity may exist between the dimensions of engagement. However, we were relieved to find that engagement as a whole did not exhibit multicollinearity with other variables, including ideal L2 self, ought-to L2 self, and enjoyment, as evidenced by the strong convergent and discriminant validity in the measurement model testing. Since our study did not investigate the influence of other variables on each dimension of engagement, we believe that the observed multicollinearity within the engagement dimensions does not significantly impact the accuracy of our findings.

**Comment 4:** The text throughout the paper requires polishing and proper copyediting.

**Reply:** Thank you for your insightful comment. We’ve invited a native English speaker to polish the language of our manuscript.

Thank you once again for your thoughtful and insightful review of our manuscript. We truly appreciate your expertise and the time you've dedicated to this process. Wishing you a wonderful day!

**Responses to Reviewer 2**

**Comments to the Author(s):**

This study addresses an important and under-researched area - the motivational and emotional factors influencing adult L2 learners' engagement in online language classes. The authors employ a robust theoretical framework, the Control-Value Theory, to examine the interplay between L2 self-guides (ideal L2 self and ought-to L2 self), enjoyment, and engagement among Chinese adult L2 learners. The sequential mixed-methods design allows for both breadth and depth in the exploration of these relationships. While the study has several strengths, there are also areas that require further clarification and development before the manuscript is ready for publication.

One of the key strengths of this study is its focus on adult L2 learners, a demographic that has received limited attention in the existing literature compared to school-aged learners. Given the distinct challenges and motivations of adult learners, such as balancing language learning with professional and personal obligations, this study fills an important gap in our understanding. The authors make a compelling case for why this population warrants special consideration in L2 motivation research.

The theoretical grounding of the study in the Control-Value Theory is another strength. The authors provide a clear rationale for how the CVT framework can elucidate the relationships between L2 self-guides, enjoyment, and engagement. The visual representation of the theoretical links (Figure 1) effectively illustrates the proposed pathways. Situating the study within this established theory enhances the conceptual clarity and interpretability of the findings.

Methodologically, the use of a sequential mixed-methods design is well-justified. The quantitative phase, with its large sample size (n=367), allows for the statistical testing of the hypothesized relationships using structural equation modeling. The subsequent qualitative phase, involving semi-structured interviews with a subset of participants, provides a deeper understanding of the statistical patterns observed. This integration of quantitative and qualitative data strengthens the validity and richness of the conclusions drawn. However, there are several areas where the manuscript could be improved.

Dear Reviewer 2,

We would like to sincerely thank you for your insightful and constructive feedback, as well as for your patience throughout the review process. We have considered each of your valuable suggestions and made the necessary revisions accordingly.

We hope that the updated version now meets your expectations. For your convenience, we have highlighted the revised sections in green in our manuscript. Once again, we deeply appreciate your expertise and kindness. Wishing you a wonderful day!

Best regards

**Comment 1:** While the authors mention that the scales used to measure L2 self-guides, enjoyment, and engagement were adapted from previous studies, more details are needed about this adaptation process. How exactly were the items modified to fit the context of adult online learners? Were any new items developed? Providing this information would enhance the replicability of the study.

**Reply:** Thank you very much for your insightful comment. To clarify, the adaptation of the scale items was aimed at making them more relevant for our participants, who are adult L2 learners, as the original scale items were designed for school students. Additionally, the original scales assessing enjoyment and engagement were intended for offline learning contexts, so our goal was to tailor the items to be more career-focused and suitable for an online learning environment. It is important to note that no new items were created during this process.

The entire adaptation was carried out collaboratively by both authors (the first author and corresponding author) through careful negotiation. Furthermore, two adult L2 learners, who are friends of the authors, were involved in this process to ensure the adapted items accurately reflected their learning experiences. In fact, the inspiration for this study came from the experiences of these friends of the authors. However, to maintain impartiality, the two adult L2 learners were not included as participants in the study.

We have provided this additional information in the following section:

## 4.2 Instruments

We would like to clarify that the adaptation of the scale items was intended to make them more relevant for our participants, who are adult L2 learners. The original scale items were designed for school students, which prompted us to modify them accordingly. Additionally, the original scales assessing enjoyment and engagement were developed for offline learning contexts, so we tailored the items to better fit a career-focused and online learning environment. It is important to note that no new items were created in the process.

The entire adaptation was carried out collaboratively by both authors, through careful discussion and negotiation. To further ensure the relevance of the adapted items, two adult L2 learners were involved in the process to confirm that the items accurately reflected their learning experiences. However, to maintain impartiality, these two adult L2 learners were not included as participants in the study.

**Comment 2:** The description of the qualitative data analysis procedures is somewhat sparse. The authors state that they followed the six-phase thematic analysis approach outlined by Braun and Clarke (2006), but do not provide much detail on how each phase was implemented. For example, how were initial codes generated? How were themes reviewed and refined? A more transparent and thorough account of the qualitative analysis process would strengthen the trustworthiness of the findings.

**Reply:** Thank you for your insightful and thoughtful comment. Apologies for the brief description. In Section 4.4 “Data Analysis”, we provide a more detailed and transparent account of the qualitative analysis process to enhance the trustworthiness of our findings, as follows:

Since the goal of the qualitative phase is to identify the motivational factors driving Chinese adult L2 learners' language learning, providing insights for the quantitative findings, the extracted themes are categorized as either "ideal L2 self" or "ought-to L2 self”. Our qualitative analysis followed an inductive approach, allowing themes to emerge naturally from the data. To enhance the repeatability and transparency of our coding process, we include an example of an interview excerpt from the results section as follows:

*“I'm focused on accessing information, crucial for my computer science background. I've come to understand the significance of English in my career, as programming languages and tools predominantly originate from Western countries. When facing technical challenges, the most reliable solutions and cutting-edge technologies are often documented in English. Relying on translations can result in inaccuracies and additional costs, whereas English resources are freely available. To bridge the information gap and stay competitive, mastering English is indispensable.”*

**Table 2.** The example for the detailed coding process.

| Procedures | Explanation |
| --- | --- |
| 1. Familiarization | The focus is on career advancement, access to information, and staying competitive in a technical field. There is a sense of practical necessity and career-related motivation. |
| 2. Initial coding | **Code 1**: “*Programming languages and tools from Western countries*” – English is essential for technical resources.  **Code 2**: “*Reliable solutions and cutting-edge technologies are documented in English*” – English is key for accessing up-to-date, accurate resources.  **Code 3**: “*Bridge the information gap and stay competitive*” – Motivation stems from the need to stay competitive by overcoming knowledge gaps. |
| 3. Searching for themes | **“Accessing information”** – The participant emphasizes the importance of English for accessing crucial resources based on codes 1, 2, and 3. |
| 4. Reviewing Themes | **“Accessing information”** is driven by the professional need to master English, making it a clear example of a “**Career-driven necessity”**. |
| 5. Defining Themes | Check if the themes align with the data and fit within the broader motivational framework (ideal vs. ought-to L2 self). Clearly, “**Career-driven necessity”** aligns with **ought-to L2 self.** |
| 6. Reporting | The interview data reveals a clear focus on career-driven necessity, namely, **ought-to L2 self**. |

**Comment 3:** The discussion of the quantitative results could be more fully developed. While the authors do an excellent job of presenting the SEM findings and relating them back to the hypotheses, there is limited exploration of why certain hypotheses were not supported. For instance, why might the ideal L2 self not significantly predict enjoyment and engagement in this context, contrary to previous studies? The authors offer some potential explanations based on the qualitative data, but a deeper engagement with the extant literature and theory would enrich the interpretation.

**Reply:** Thank you for your insightful and thoughtful comment. When we were drafting our literature review and discussion sections, we aimed to include existing research on the motivation of adult second language learners. To do this, we conducted a search in two prestigious databases, Web of Science and Scopus, using the following keywords: "adult second language learners motivation," "adult English learners motivation," "workers second language motivation," "workers English motivation," "second language motivation workplace," and "English motivation workplace." Unfortunately, we were unable to find any relevant articles. The search results primarily yielded studies on school-aged L2 learners’ motivation. We believe that this gap in the literature highlights the value of our study.

**Comment 4:** The practical implications of the study could be more clearly articulated. Given the findings about the importance of the ought-to L2 self for adult learners' enjoyment and engagement, what specific strategies might online language programs employ to leverage this? How can course designers and instructors create environments that cater to the unique motivations of adult learners? Providing more concrete recommendations grounded in the study's insights would enhance the applied value of the research.

**Reply:** Thank you for your insightful and thoughtful comment. In response to your feedback, we have outlined five strategies that may assist course designers and instructors in creating learning environments that effectively address the unique motivations of adult learners. These strategies are as follows:

From a practical standpoint, to effectively leverage the concept of ought-to L2 self in online language programs, course designers and instructors need to consider the unique motivations of adult learners. These learners often have specific, pragmatic reasons for learning a second language (such as career advancement, travel, or meeting professional obligations), so creating an environment that aligns with these motivations is crucial for enhancing their enjoyment and engagement. Here are some specific strategies:

*1. Incorporating real-world application and career-relevant content*

To enhance learner motivation, course content should be directly tied to real-world scenarios or professional contexts, highlighting how language proficiency can improve job performance and career opportunities. This can be achieved by incorporating case studies, role-playing exercises, industry-specific vocabulary, and problem-solving tasks that reflect the learner’s work environment. For instance, an online English program tailored for healthcare professionals might include medical terminology, communication strategies for patient interaction, and simulations of real-life situations. By aligning course materials with learners' professional responsibilities or career goals, instructors help them visualize how language learning connects to their ought-to L2 self—the expectations they feel from work, family, or society. This alignment makes the learning process more personally relevant and motivating, as learners see a clear link between their language skills and their broader life objectives.

*2. Setting clear and achievable goals with progress tracking*

Establishing clear, measurable learning goals is essential for helping learners track their progress in meaningful ways. These goals should align with learners' external motivations, enabling them to visualize the steps needed to achieve their professional or personal aspirations. To support this, tools such as progress dashboards, badges, or certificates can be used to mark milestones, while encouraging learners to set specific personal language goals—such as mastering business-related phrases, passing a certification exam, or improving communication with colleagues. Celebrating these achievements reinforces their sense of accomplishment. Adult learners are often motivated by tangible outcomes, and setting clear goals not only provides a roadmap for success but also helps them feel accountable to both themselves and the external expectations tied to their careers or job requirements.

*3. Fostering social interaction and community building*

Creating opportunities for learners to engage in real-life communication is a powerful strategy, especially for adult learners who view language as a tool for social or professional networking. This can be achieved by facilitating group discussions, peer reviews, collaborative projects, or live virtual sessions where learners participate in conversations or debates relevant to their field of work. Additionally, learners can be encouraged to form study groups or discussion forums where they can share professional experiences and language-learning tips. Social interaction not only reinforces the idea that language is a means to connect with others—such as colleagues, clients, or the broader professional community—but also helps learners build their ought-to L2 self, motivating them to meet the expectations of their professional or social networks.

*4. Integrating personalized feedback and support*

Providing personalized, constructive feedback is crucial for addressing learners' specific needs, challenges, and progress toward their goals. Instructors can offer feedback that is both encouraging and practical, helping learners understand how their current language proficiency supports their professional or personal aspirations. For example, feedback like, “You’ve done a great job using technical terms in your presentation. With a bit more work on your pronunciation, you’ll feel even more confident in client meetings,” highlights both strengths and areas for improvement. Personalized feedback demonstrates that the instructor understands the learner’s goals and expectations, which can significantly enhance the learner's motivation to work toward their ought-to L2 self. It also reinforces the idea that the course is not just about language acquisition, but about achieving tangible, practical results in their daily lives.

*5. Creating a flexible learning environment*

Offering a flexible learning structure is essential for adult learners who need to balance their studies with work and other responsibilities. To support this, instructors can provide asynchronous learning options, such as videos, readings, and self-paced assignments, allowing learners to access content on their own schedules. Additionally, offering synchronous sessions at varying times accommodates different time zones or work commitments. This flexibility helps adult learners stay motivated without feeling overwhelmed, enabling them to meet both their professional development or personal goals and their other responsibilities. By catering to their ought-to L2 self, this approach ensures that learners can fulfill external expectations while maintaining a healthy work-life balance.

**Comment 5:** In terms of minor issues, there are a few instances of awkward phrasing or grammatical errors (e.g., "Our study collected quantitative data during summer from three universities in China that offer multilingual online training for adult L2 learners" could be rephrased for clarity). A thorough round of proofreading and editing would help polish the writing.

**Reply:** Thank you very much for your thoughtful and insightful comment. We have carefully proofread the manuscript to enhance its overall flow, with particular attention given to improving the clarity and coherence of the abstract.

Overall, this study makes a valuable contribution to our understanding of the motivational dynamics underlying adult L2 learners' engagement in online classes. The focus on an under-researched population, the solid theoretical foundation, and the mixed-methods approach are notable strengths. However, the manuscript would benefit from more methodological detail, a deeper discussion of the findings in light of prior research, and clearer practical implications. With some revisions to address these points, the paper has the potential to make a significant impact in the field of L2 motivation.

Dear reviewer 4, thank you for your expertise and kindness in reviewing our manuscript. We can clearly see that, following your insightful comments, the rigor and depth of our work have been greatly enhanced. We sincerely appreciate your valuable input. Thank you once again, and have a wonderful day!
